# Oral Molnupiravir and Nirmatrelvir/Ritonavir for the Treatment of COVID-19: A Literature Review with a Focus on Real-World Evidence

**DOI:** 10.3390/idr15060061

**Published:** 2023-10-25

**Authors:** Ioannis Karniadakis, Nikolaos Mazonakis, Constantinos Tsioutis, Michail Papadakis, Ioulia Markaki, Nikolaos Spernovasilis

**Affiliations:** 1 Cardiff Transplant Unit, University Hospital of Wales, Cardiff and Vale University Health Board, Cardiff CF14 4XW, UK; ioannis.karniadakis@wales.nhs.uk; 2 Internal Medicine Department, Thoracic Diseases General Hospital Sotiria, 11527 Athens, Greece; nikosmazwnakis13@hotmail.gr (N.M.); tzouliamar95@gmail.com (I.M.); 3 School of Medicine, European University Cyprus, 6 Diogenis St., Nicosia 2404, Cyprus; 4 3rd Internal Medicine Department & Diabetes Center, General Hospital of Nikaia Piraeus “Agios Panteleimon”, 18454 Piraeus, Greece; papadakis.mixal@gmail.com; 5 Department of Infectious Diseases, German Oncology Center, Agios Athanasios, Limassol 4108, Cyprus; nikolaos.spernovasilis@goc.com.cy

**Keywords:** COVID-19, pandemic, SARS-CoV-2, oral antivirals, Paxlovid, ritonavir, nirmatrelvir, molnupiravir

## Abstract

Vaccines remain the cornerstone of medical prevention and are highly effective in reducing the risk of severe disease and death due to coronavirus disease 2019 (COVID-19). In the context of expanding the therapeutic armamentarium against COVID-19, molnupiravir (Lagevrio) and ritonavir-boosted nirmatrelvir (Paxlovid) were developed, constituting the first effective oral treatments against severe acute respiratory syndrome coronavirus 2 (SARS-CoV-2). In this narrative review, we retrospectively inquired into the clinical trials and real-world studies investigating the efficacy of these agents. Overall, clinical trials and real-world studies have demonstrated the efficacy of both agents in reducing hospitalization and death rates in COVID-19 patients. As per current recommendations, their use is suggested in patients with mild to moderate symptoms who are at high risk of developing severe disease. Nevertheless, limited data exist regarding their efficacy in specific subpopulations, such as immunocompromised patients, those with severe kidney disease, pregnant women, and children.

## 1. Introduction

Vaccination against COVID-19 has been established as the most valuable weapon in preventing severe systemic disease and subsequent mortality, as demonstrated using clinical trials and real-world data [1,2]. However, a significant proportion of the global population remains unvaccinated, vaccine-induced immunity wanes over time, not all vaccinated people mount an adequate immune response, and new SARS-CoV-2 variants maintain the ability to circumvent immunity conferred by natural infection or vaccination [3,4,5,6].

An array of effective antiviral medications is vital in reducing morbidity and mortality caused by SARS-CoV-2 infections [2]. Rationally, new oral antivirals targeting parts of the virus other than spike protein could play a complementary role in reducing patient hospitalization and death rates regardless of vaccination status, time from vaccination or the patient’s immune state. 

Pfizer’s Paxlovid [nirmatrelvir (PF-07321332); ritonavir] and molnupiravir (Lagevrio EIDD-2801) by Merck and Ridgeback Biotherapeutics are the first effective oral treatments that are approved for use against SARS-CoV-2 [7]. This narrative review aims to summarize the current knowledge regarding the new oral antivirals, focusing on their mechanism of action and discussing the main findings from clinical trials that granted them an Emergency Use Authorization (EUA). Emphasis is placed on subsequent real-world studies, which assessed their safety and efficacy in large cohorts of patients beyond the strict protocols of clinical trials. 

## 2. Oral Antivirals

SARS-CoV-2 utilizes an RNA-dependent RNA polymerase (RdRp) with replicative and transcriptive capacity for survival [8]. Viral polymerases constitute an ideal target for coronavirus-specific treatments. It is important to highlight, however, that coronaviruses are innately resistant to nucleoside analog treatments that suspend the physiologic RNA elongation process due to the proof-reading activity facilitated with the RdRp enzyme [9]. 

### 2.1. Molnupiravir (EIDD-2801)

Molnupiravir (Lagevrio or EIDD-2801) represents a prodrug of β-D-*N*^4^-hydroxycytidine (NHC) that demonstrates higher than 100-fold potency against SARS-CoV-2 in comparison with ribavirin and favipiravir [10]. Its efficacy in limiting replication has been documented in numerous viruses beyond SARS-CoV-2: influenza viruses [11], Venezuelan equine encephalitis virus [12], respiratory syncytial viruses [13], Chikungunya virus [14], Ebola virus [15] and hepatitis C virus (HCV) [16]. 

**Figure 1:**
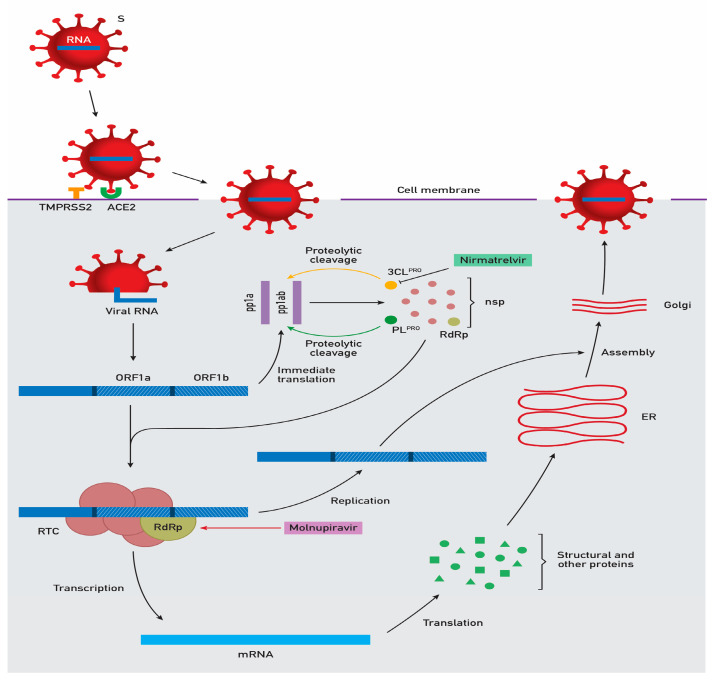
Mechanism of action of nirmatrelvir and molnupiravir during the life cycle of SARS-CoV-2.

Regarding its efficacy against SARS-CoV-2, molnupiravir has been demonstrated to exert various effects in vivo. Wahl et al. demonstrated that molnupiravir could reduce viral replication in human lung tissue, regardless of whether it was administered in prophylactic or therapeutic regimens [17]. Similarly, Cox et al. documented a substantial viral load reduction and suppressed contamination in the upper respiratory system of ferrets treated with molnupiravir [18]. These observations led to intense scientific efforts to elucidate molnupiravir’s mechanism of action against the SARS-CoV-2 RdRp (Figure 1).

The spike (S) protein on the surface of viral particles binds to the angiotensin-converting enzyme 2 (ACE2) cell-surface receptor. The transmembrane serine protease 2 (TMPRSS2) is necessary for priming the S protein. These interactions lead to the fusion of the viral and cellular membranes, promoting viral entry. After the release of the genome into the cytoplasm, an immediate translation of two regions called open reading frames 1a and 1b (ORF1a and ORF1b) takes place, producing two polypeptides, pp1a and pp1ab. Sixteen non-structural proteins are released from these polypeptides upon proteolytic cleavage via the papain-like protease (PL^PRO^) and the 3-chymotrypsin-like protease (3CL^PRO^). Among these non-structural proteins are the RNA-dependent RNA polymerase (RdRp) and other enzymes, which constitute the viral replication and transcription complex (RTC) and are essential for RNA synthesis. Messenger RNAs (mRNAs) are translated into structural and other proteins, and viral assembly occurs at the endoplasmic reticulum (ER)-to-Golgi compartment. After this process, viral particles are ready to exit the infected cell. Nirmatrelvir inhibits 3CL^PRO^, blocking the viral proliferative process, and molnupiravir interferes with RdRp, causing “lethal mutagenesis”.

There is a two-step process that results in the molnupiravir-driven “lethal mutagenesis” of SARS-CoV-2 [19]. In more detail, RdRp incorporates β-d-*N*^4^-Hydroxycytidine (NHC) monophosphate (M) nucleotides into the elongating molecule instead of nucleotides C or U. Subsequently, the NHC-carrying RNA template drives the production of mutated end-products with confined functionality [19]. As outlined above, it is essential to override the proofreading activity of the RdRp [20], and molnupiravir exhibits adequacy. 

NHC nucleotides have been demonstrated to compromise the coronavirus’s innate mechanism of survival mutation development. In 2019, Agostini et al. investigated the effects of NHC on the inhibition and development of resistance in two distinct coronaviruses, the Murine Hepatitis Virus (MHV) and the Middle East Respiratory Syndrome coronavirus (MERS-CoV) [21]. NHC mediated a mutagenic mechanism of virus inhibition, which concurrently established an accumulated genetic mutation load sufficient to impede viral resistance development [21].

On the other hand, a wave of skepticism emerged over whether this mutagenesis could also collaterally affect the host human cells. In a side-by-side comparison of the mutagenic effects of three distinct ribonucleoside analogs against SARS-CoV-2 conducted by Zhou et al., the authors suggested that even a brief treatment regimen with these agents would likely also inadvertently expose the host to their mutagenic properties [10]. Simultaneously, a panel of FDA experts recently emphasized the risk of teratogenesis as well as the possible emergence of viral strains resistant to the current treatment approaches [22].

### 2.2. Paxlovid [Nirmatrelvir (PF-07321332); Ritonavir]

The genomic analysis of SARS-CoV-2 has demonstrated that the viral RNA codes for four structural proteins (one of which is the spike protein) and two polypeptides (pp1a and pp1ab) [23]. Through protease-based proteolytic cleavage, these polypeptides are consequently further processed into smaller proteinic molecules (helicase, single-stranded RNA-binding protein, RdRp, 2′-*O*-ribose methyltransferase, endoribonuclease and exoribonuclease), which demonstrate a key role in viral replication [23]. 

The 3-chymotrypsin-like protease (3CL^PRO^) is the central protease facilitating the process mentioned above and assumes a vital role in viral replication and survival [24,25]. It is a proteinic molecule with 306 amino acid residues forming a homodimer, each unit of which consists of three domains (I, II and III). The active site of this protease, containing the catalytic dyad of Cys145-His41, is located between its domains I and II [26]. Demonstrating a central role in the viral proliferative process, in combination with having no homology to human proteases, 3CL^PRO^ has emerged as a potential target of precision antiviral medicine [25].

Protease inhibitors already exist in the therapeutic arsenal against other viral infections, including HIV infection and hepatitis C [24], with drug-repurposing processes being appealing in a global pandemic setting. 

PF-07321332 (nirmatrelvir) is an oral medication developed from compounds refined during the SARS-CoV-1 epidemic, and it was designed for precise 3CL^PRO^ inhibition. This achievement was deemed possible since the main proteases of SARS-CoV-1 and SARS-CoV-2 exhibit a high sequence of substrate binding site homology [25]. Nirmatrelvir boasts proven in vitro and in vivo antiviral activity mediated through the blockade of the viral proteolytic cleavage cascade (Figure 1) [25]. 

In terms of pharmacokinetics, nirmatrelvir has been demonstrated to be metabolized through the CYP3A4 enzyme, and thus, co-administration with a CYP3A4 inhibitor could augment its therapeutic potential [24]. Indeed, the strategic co-administration of nirmatrelvir with ritonavir (Paxlovid), a known CYP3A4 inactivator, has been suggested, inspired by previous drug co-administrations that were used in a similar fashion against HIV [27].

## 3. Evidence beyond Clinical Trials

On 11 October 2021, Merck, in collaboration with Ridgeback Biotherapeutics, announced the submission of their application to the U.S. Food and Drug Administration (FDA) for the Emergency Use Authorization (EUA) of molnupiravir [2]. Their submission was supported by promising results from an interim analysis of the phase 3 MOVe-OUT clinical trial conducted on non-hospitalized adult patients with mild to moderate COVID-19 identified as being at increased risk for disease progression. They included patients of both sexes with the following criteria: onset of symptoms ≤5 days prior to the day of randomization and at least one predisposing factor for increased risk for severe illness. Patients that were excluded were pregnant and breastfeeding women, patients with severe renal failure or on dialysis, HIV-positive patients with a recent viral load of >50 copies/mL and patients with a history of a hepatitis B virus (HBV) or HCV infection with concomitant hepatic impairment [28]. The researchers demonstrated that a 5-day course of molnupiravir 800 mg twice daily reduced the risk of hospitalization or death by 48% [29]. This oral agent had already been authorized in the UK [30]. 

On 26 November 2021, the two companies updated on the results of the MOVe-OUT study, eventually demonstrating a relative risk reduction of 30% in hospitalization or death and an adverse effect profile consistent with the one on the interim analysis [31,32]. Finally, on 23 December 2021, the FDA announced an EUA for molnupiravir for the treatment of adults with mild to moderate COVID-19 at high risk for progression to severe disease when alternative therapeutic options are unavailable or clinically irrational. A total of forty capsules (four 200 mg capsules twice daily for five consecutive days) would be administered within five days of symptom onset [32,33]. According to secondary and post hoc analyses of patients’ self-reported symptoms in the MOVe-OUT trial, sustained resolution or improvement of symptoms was more likely and disease progression was less probable in the molnupiravir versus placebo group of participants [34]. 

Molnupiravir was also evaluated in the MOVe-AHEAD study for post-exposure prophylaxis, assessing its efficacy in preventing laboratory-confirmed symptomatic infection in adults in close contact with a person positive for SARS-CoV-2 with symptoms [35]. However, on 21 February 2023, Merck announced that the reduction in the risk of infection was not statistically significant after using molnupiravir [36]. Furthermore, molnupiravir was tested in hospitalized patients with COVID-19 in the MOVe-IN trial, demonstrating no clinical benefit compared to placebo [37].

During the emergence of the Omicron variant, PANORAMIC, a UK-based multi-center randomized controlled trial, evaluated the effectiveness of the addition of molnupiravir to the usual treatment of COVID-19 in high-risk outpatients, defined as being ≥50 years of age or ≥18 years with relevant comorbidities. In contrast to the MOVe-OUT study, this open-label trial did not demonstrate a significant reduction in hospitalization or death in the group of patients treated with molnupiravir plus standard care compared to standard care alone [38]. Three months later, Malin et al. published the findings of a systematic review of nine randomized controlled trials, each of which compared patient outcomes after molnupiravir administration ± standard care versus standard care and/or placebo. In agreement with PANORAMIC, the authors demonstrated that molnupiravir did not affect mortality and hospitalization rates in the largely immunized general population of COVID-19 outpatients [39].

On the other hand, Xie et al., using information collected from electronic health records, reproduced the outcomes of a randomized controlled trial (RCT) designed to evaluate the benefit of molnupiravir administration in comparison with no treatment in adult patients at high risk of developing severe COVID-19 disease in the community. The authors suggested that molnupiravir might reduce hospital admission rates or death rates at 30 days compared to no treatment [40]. In addition, a large US cohort study of 229,286 patients demonstrated that in SARS-CoV-2-positive individuals with at least one risk factor for progression to severe disease, molnupiravir administration was associated with a decreased risk of post-acute sequelae compared to no treatment, regardless of vaccination status and history of prior infection [41].

To address the disparities surrounding the efficacy of molnupiravir in the literature, Tian et al. conducted a systematic review and meta-analysis of nine RCTs that included 31,573 COVID-19 patients. The researchers demonstrated that the molnupiravir group of patients exhibited higher rates of clinical improvement on day 5 of treatment, but no significant difference was noted in terms of mortality, hospitalization or adverse event rates between the two groups [42]. In contrast, another systematic review and meta-analysis of nine RCTs demonstrated that molnupiravir administration was associated with a lower risk of mortality and hospitalization among non-hospitalized patients [43].

In a more recent systematic review and meta-analysis of fourteen RCTs with a yield of 34,570 adult patients with non-severe COVID-19, which aimed to evaluate the efficacy of molnupiravir administration, it was outlined that the antiviral agent was weakly associated with a reduction in the hospitalization risk. Still, no significant differences were indicated in terms of adverse event occurrence, all-cause mortality, length of hospital stay, risk of mechanical ventilation and time-to-symptom resolution [44].

Of note, molnupiravir gathered substantial attention regarding its potential efficacy against severe COVID-19 disease and associated sequelae in unvaccinated individuals in clinical studies such as the MOVe-OUT clinical trial. However, further investigation in studies such as the PANORAMIC RCT and subsequent systematic reviews and meta-analyses failed to demonstrate a strong association between molnupiravir administration and a reduction in the already-low hospitalization and all-cause mortality rates in high-risk but previously vaccinated individuals. 

On the other hand, Pfizer’s Paxlovid (ritonavir-boosted nirmatrelvir) was administered to unvaccinated high-risk adult outpatients with mild to moderate disease in the context of phase 2/3 of the EPIC-HR study. EPIC-HR eligibility and exclusion criteria were similar to those used in the MOVe-OUT trial for molnupiravir [45]. The final results indicated that a five-day regimen of Paxlovid reduced the risk of hospitalization or death by 89% in patients treated within three days of symptom onset [46]. On 22 December 2021, the FDA announced an EUA for this oral drug for the treatment of adult and pediatric patients (12 years of age and older, with at least 40 kg body weight) with mild to moderate symptoms who are identified as being at increased risk for disease progression [47]. 

In parallel, the EPIC-SR study in standard-risk patients primarily evaluated time to sustained symptom alleviation using Paxlovid within 3 or 5 days of symptom onset. This clinical trial also included a proportion of vaccinated participants with medical conditions predisposing them to severe disease [48]. Preliminary reports indicated that the primary outcome of symptom relief failed to reach its prespecified endpoint, and a 70% reduction in hospitalization and no deaths were observed in the population treated with Paxlovid [49]. Finally, however, the study demonstrated no significant decrease in the rates of hospitalization or death [50]. Moreover, in both the EPIC-HR and the EPIC-SR studies, participants in the treatment arm had an approximately 10-fold decrease in viral load on day 5 of treatment [49]. Furthermore, a phase 2/3 study for post-exposure prophylaxis (EPIC-PEP) was initiated in September 2021, assessing the efficacy of Paxlovid in preventing the development of symptomatic COVID-19 infection after exposure to a person with confirmed symptomatic infection [51]. However, non-significant results were announced on 29 April 2022 [52]. Finally, the EPIC-Peds study is ongoing, recruiting non-hospitalized pediatric patients with mild to moderate COVID-19 symptoms at high risk for disease progression and assessing Paxlovid’s safety and efficacy in this subset of patients [53].

To elucidate the effectiveness of nirmatrelvir during the era of the Omicron variant, Arbel et al. conducted a study with a sample population size of nearly 110,000 patients over 40 years of age, 4% of whom were treated with Paxlovid. It was indicated that among patients over 65 years of age, hospitalization and overall mortality rates were significantly lower in the Paxlovid treatment cohort, with no similar benefit demonstrated in younger patients [54]. 

Najjar-Debbiny et al. also conducted a study during the Omicron era, including more than 180,000 adult patients identified as being at risk for severe COVID-19. Of these patients, 2.6% were treated with Paxlovid, and 75.1% had adequate COVID-19 vaccination status at the time. The researchers demonstrated that both Paxlovid and complete COVID-19 vaccination status were independently associated with reduced morbidity and increased overall survival. As a secondary outcome, it was indicated that Paxlovid may be more beneficial in frail patients who belong to certain risk groups, including the elderly, the immunocompromised, and patients with a complicated cardiovascular or neurological background [55]. Indeed, a retrospective cohort study of real-world data in Singapore indicated that outpatient Paxlovid use was beneficial amongst the vaccinated elderly but did not significantly reduce the already low risk for severe COVID-19 in a highly vaccinated outpatient population [56].

To expand upon the previous studies, Ganatra et al. elaborated on the benefits of Paxlovid in high-risk vaccinated adult individuals in the outpatient setting, conducting a retrospective study of two separate cohorts with 1130 patients each, based on Paxlovid administration status. The researchers demonstrated that treatment with Paxlovid in this patient population was associated with decreased rates of ER visits, hospitalization, complications, resource expenditure and death [57]. Complementarily, a large US cohort study was designed to investigate the effect of nirmatrelvir administration on the incidence of post-COVID-19 conditions (PCC or long COVID); the authors concluded that individuals with an acute viral infection and at least one risk factor for severe disease progression were less likely to progress to long COVID after treatment with nirmatrelvir, irrespective of history of prior infection or vaccination status [58]. PCC, or long COVID, defined as the persistence of symptoms for months beyond the acute infection episode, is of significant concern due to its long-standing health and social implications, and the reason is twofold: firstly, due to the considerable number of SARS-CoV-2 infections, and secondarily, due to its high incidence, estimated to be up to 17% of acute infections in the UK [59]. Current evidence suggests an association between PCC incidence and disease severity during the phase of initial infection [60]. Arguably, the preventative role of nirmatrelvir in PCC occurrence may be an eventuality of its protective effect against progression to severe disease. However, further research is warranted to establish such an association.

Finally, an emulated randomized control trial by Xie et al. demonstrated that in patients with COVID-19 at risk of severe disease, nirmatrelvir administration was associated with reduced rates of hospitalization or 30-day mortality compared to no treatment, regardless of vaccination or history of previous infection status [61]. Similarly, real-world data from two large independent matched observational cohort US-based studies indicated that treatment with Paxlovid reduced the risk of hospitalization and all-cause mortality in COVID-19-positive outpatients [62,63].

To evaluate the effect of either molnupiravir or Paxlovid in patients with mild to moderate COVID-19 and at least one underlying disease, Wai et al. conducted a retrospective study of 54,355 individuals in Hong Kong who were prescribed either one of the above antiviral agents in an inpatient or outpatient setting. The researchers demonstrated that oral antiviral agents were associated with a significantly decreased hazard of all-cause mortality with good safety profiles. Secondarily, it was indicated that any oral antiviral prescription was associated with fewer hospitalizations, and shorter lengths of stay, thereby significantly reducing hospital costs [64]. Concurrently, two retrospective cohort studies by Lui et al. and Wu et al. investigating the effect of oral antivirals on non-hospitalized patients with Diabetes Mellitus type II (DMII) and COVID-19 concluded that the antiviral agents were associated with decreased rates of all-cause mortality and hospitalization in DMII outpatients [65,66].

Cegolon et al. published a retrospective cohort study in May 2023 in which they investigated the clinical outcomes of molnupiravir, Paxlovid and Sotrovimab against standard-of-care controls at three distinct endpoints (hospitalization at 30 days, mortality at 30 days and time between COVID-19 diagnosis and first negative swab test result) in a total number of 386 COVID-19 outpatients [67]. The authors concluded that not only did all three aforementioned antiviral agents prove to be more effective than the current standard of care in preventing hospital admission and/or death secondary to SARS-CoV-2, but they also reduced time to a negative RT-PCR swab test. Interestingly, as a secondary study outcome, it was indicated that the number of vaccinations had a protective effect when it came to hospitalization [67]. 

Concerning hospitalized patients with COVID-19, Wan et al. conducted a target trial emulation study investigating adult inpatients who received either molnupiravir or Paxlovid during the Omicron outbreak. The researchers concluded that molnupiravir and Paxlovid reduced all-cause mortality rates within 28 days of admission but did not affect the risk of ICU admission or the need for mechanical ventilation [68].

Notably, many clinical trials and real-world studies almost universally included patients identified as high risk for disease progression, effectively excluding vulnerable individuals who had already reached severe COVID-19 status. On this front, Liu et al. designed a multicenter randomized control trial that included adult patients infected with SARS-CoV-2, hospitalized within the previous 48 h with a SOFA/Charlson score ≥ 2, with concomitant, already identified severe comorbidities. The authors endeavored to assess the safety and efficacy of Paxlovid in the described patient population and defined severe comorbidities as including but not limited to immunosuppressed status, Chronic Obstructive Pulmonary Disease (COPD), hypertension complicated with associated target organ injury, both acute and chronic cardiac insufficiency and renal failure. This study concluded that treatment with Paxlovid did not improve patient outcomes in terms of all-cause mortality on day 28 as well as SARS-CoV-RNA burden in the aforementioned patient group [69]. On the other hand, a territory-wide study conducted by Wai et al. demonstrated that among COVID-19 hospitalized patients, the use of either Paxlovid or molnupiravir was associated with a significantly lower incidence of -related mortality and sepsis against no treatment controls [70]. 

Comparing the efficacy of Paxlovid against molnupiravir, Amani et al. conducted a systematic review and meta-analysis including eighteen studies with a 57,659 patient yield, indicating the superiority of Paxlovid in reducing all-cause mortality, hospitalization rates and negative polymerase chain reaction conversion times over molnupiravir. However, no significant difference was outlined between the two antiviral agents in terms of COVID-19 rebound after treatment [71]. Similarly, a real-life comparison cohort study of 17,977 patients sampled from the general population concluded that Paxlovid was superior to molnupiravir in reducing the risk of mortality by day 28, regardless of vaccination status and underlying comorbidities [72]. In contrast, in a retrospective review of 3207 high-risk COVID-19 outpatients, Paxlovid and molnupiravir produced similar results in reducing all-cause hospitalization rates [73]. 

To examine the rate and relative risk of COVID-19 rebound after completion of treatment, Wang et al. conducted a retrospective cohort study of more than 13,000 patients infected with COVID-19 and subsequently treated with Paxlovid or molnupiravir. COVID-19 rebound was defined as either COVID-19 infection, COVID-19-associated symptoms, or hospitalization. What was demonstrated was that patients who experienced COVID-19 rebound had a significantly higher frequency of concomitant medical conditions and that the rebound risk was similar for both agents under review [74]. More recently, Wong et al. compared the SARS-CoV-2 viral burden rebound rates among two cohorts of patients: one that received either Paxlovid or molnupiravir as treatment and the other that received no treatment. The authors determined that the viral burden rebound rates were similar between the two cohorts and that viral burden rebound was not associated with any clinical adverse events [75]. COVID-19 rebound incidence and associated risk factors have not been extensively investigated in the literature, especially in the setting of oral antiviral administration. Perelson et al. discussed that a 5-day course of Paxlovid may lead to incomplete viral clearance facilitated by incomplete target cell depletion, which was demonstrated to remain at numbers sufficient to cause disease rebound. Conversely, the authors suggest that a modification of the oral antiviral treatment duration could play a significant role in reducing COVID-19 rebound occurrence [76].

## 4. Considerations in Clinical Practice

### 4.1. Drug Resistance and Drug Interactions

The challenges that are imposed by oral antiviral regimen administration in the real clinical setting, beyond strict clinical trial administration and adherence to treatment protocols, are vital to be discussed. Despite the complete duration of the oral antiviral regimen being merely five days, the total number of pills that must be ingested daily may affect appropriate compliance [77]. As a result, it could be argued that inconsistent adherence to the prescribed regimen may assume a causative role in the emergence of induced viral resistance. 

Various references describing the development of resistance to protease inhibitors have been reported in the literature in the context of antiretroviral agent use in the therapeutic management of HIV, including ritonavir [78,79]. In a recent study published by Ip et al. in 2023, the authors investigated the worldwide prevalence of mutant SARS-CoV-2 strains that demonstrated resistance to the protease inhibitors nirmatrelvir or ensitrelvir, secondary to a mutated 3CL^PRO^. They concluded that currently, one year after the approval of Paxlovid, protease inhibitor resistance prevalence remains scarce. More specifically, of the 13,446,588 3CL^PRO^ coding sequences investigated, merely 0.5% contained at least one mutation that granted resistance to the inhibitory properties of nirmatrelvir or ensitrelvir [80]. 

No substantial risks for clinically relevant drug-to-drug interactions have been identified with molnupiravir [81]. In contrast, on the basis that nirmatrelvir is known to be metabolized with P450 CYP3A, Paxlovid administration to the general population beyond a clinical trial may pose a significant challenge. Suitable patient education may be necessary regarding the potential drug-to-drug interactions of nirmatrelvir, which include antiarrhythmic medications such as amiodarone and lidocaine; anticoagulants such as warfarin, rivaroxaban and dabigatran; macrolide antibiotics; antihypertensives such as amlodipine, nifedipine and diltiazem; corticosteroids such as budesonide and methylprednisolone as well as severe life-threatening adverse interactions with calcineurin inhibitors, e.g., tacrolimus [82]. The counseling of the patients regarding nirmatrelvir interactions should also include certain over-the-counter medications, recreational drugs and herbals [83]. Concurrently, ritonavir, the second pharmaceutical component of Paxlovid, constitutes a potent P450 CYP3A inhibitor and may consequently exaggerate the existing drug interactions of nirmatrelvir [82]. Larsen et al. demonstrated that in Denmark, there exists significant potential for noteworthy Paxlovid-induced drug-to-drug interactions in the elderly population, whose underlying comorbidities, by definition, predispose them to severe COVID-19 disease [84].

### 4.2. Immunocompromised Patients

Through various cases in the literature, it has been suggested that immunodeficient individuals may harbor the SARS-CoV-2 virus for extended periods of time [77]. In a real-life study of 35 Chinese immunocompromised patients with COVID-19, Paxlovid administration within five days of diagnosis led to a more rapid clearance of viral load and a faster elimination of SARS-CoV-2 [85]. In a similar context, Graziani et al. described three cases of relapsing COVID-19 in patients receiving anti-CD20 therapy who were treated with ritonavir-boosted nirmatrelvir with favorable clinical, radiological and virological outcomes [86]. The EPIC-IC study is also currently ongoing, evaluating the efficacy of Paxlovid in immunocompromised symptomatic patients with COVID-19 who are at least 12 years of age [87]. Additionally, a post hoc analysis of the immunocompromised patient cohort of the MOVe-OUT trial demonstrated the superiority of molnupiravir compared to placebo in reducing the risk for hospitalization or death by day 29. However, this was a small subset of patients with no significant heterogeneity in the type of immune compromise [88].

In the context of hematological malignancy, Spiliopoulou et al. conducted a prospective study investigating the effect of molnupiravir and Paxlovid on COVID-19-associated infection severity and mortality in patients with multiple myeloma (MM) [89]. This subgroup of patients faces the challenge of multi-layer immune compromise, originating from treatment-induced immunosuppression on top of the natural pathophysiology of the disease. Promisingly, the authors concluded that both antivirals appear to hold potential benefits in mitigating severe SARS-CoV-2 infection outcomes among patients diagnosed with MM [89].

More recently, Salmanton-Garcia et al. conducted a head-to-head comparison of the efficacy of molnupiravir against Paxlovid in a high-risk patient population with an underlying hematological malignancy. The researchers investigated two cohorts of 116 patients that either received molnupiravir or Paxlovid for the treatment of COVID-19, while matching the patients based on sex, age and hematological malignancy severity. This match-paired study indicated that both antiviral agents had comparable efficacy in terms of hospitalization rates and overall mortality in this subgroup of patients [90].

Concerning the field of organ transplantation, a study in solid organ transplant recipient outpatients with COVID-19 demonstrated that patients who received molnupiravir had lower rates of hospitalization within 30 days compared to those who received no treatment [91]. In contrast, a study in kidney transplant recipients infected by SARS-CoV-2 exhibited no statistically significant reduction in hospitalization rates in the subgroup of patients treated with molnupiravir [92]. 

Nevertheless, more research is warranted to further clarify the role of oral antiviral treatment and the possible prophylaxis of immunodeficient SARS-CoV-2-positive individuals with or without other severe concomitant comorbidities treated in the outpatient setting.

### 4.3. Patients with Severe Chronic Kidney Disease (CKD)

NHC is not eliminated through renal clearance, and some studies have shown the favorable adverse effect profile and clinical efficacy of molnupiravir at a standard dosage of 800 mg twice daily for five days in patients with COVID-19 and advanced CKD or undergoing hemodialysis [93,94]. A retrospective cohort study in adult hospitalized patients with COVID-19 and chronic kidney disease with eGFR ≥ 30 mL/min/1.73 m^2^ revealed that early initiation of Paxlovid was associated with reduced rates of all-cause death, invasive ventilation and ICU admission [95]. Patients with eGFR < 30 mL/min/1.73 m^2^ were excluded from the initial EPIC-HR study of Paxlovid, and this oral antiviral is not recommended in this subset of patients [45]. However, Hiremath et al. suggested a modified low-dose regimen of nirmatrelvir/ritonavir for patients with advanced CKD undergoing hemodialysis, which was safe and well-tolerated [96].

### 4.4. Pregnancy and Lactation

Molnupiravir is not administered to pregnant and breastfeeding women, as they were excluded from the initial MOVe-OUT trial because of its possible mutagenic effects in mammalian cells [10,28]. These women were also excluded from the EPIC-HR study of ritonavir-boosted nirmatrelvir [45]. However, in a small study by Loza et al. in seven pregnant patients with COVID-19, Paxlovid was well tolerated, and no adverse fetal events were observed. Only one patient stopped treatment because of the development of dysgeusia [97]. Moreover, ritonavir, as part of antiretroviral therapy for pregnant women with HIV, has been extensively studied without safety or efficacy concerns [98]. Larger studies in pregnant and breastfeeding women are warranted, as current recommendations from different major international societies regarding the use of Paxlovid during pregnancy and lactation are still conflicting [99].

### 4.5. Children

Currently, there is anecdotal evidence of the efficacy of oral antiviral agents in children diagnosed with COVID-19. In 2022, Yan et al. investigated the effectiveness of Paxlovid in five children with underlying diseases, using age-matched patients with comorbidities as controls. In the study, all child patient cases recovered from the disease, but no difference was demonstrated in terms of viral shedding times [100]. Another study conducted by Li et al. explored the clinical efficacy of Paxlovid in children with a baseline hematological malignancy who were diagnosed with a SARS-CoV-2 infection. Among the Paxlovid and non-Paxlovid cohorts, it was indicated that children who received the oral antiviral treatment demonstrated shorter times of viral clearance and no adverse reactions to treatment, but on the other hand, underwent more extended periods of fever and presented with higher inflammation markers [101].

## 5. Current Recommendations according to the National Institutes of Health (NIH)

On 25 May 2023, Paxlovid was approved by the FDA for the treatment of non-hospitalized adult patients with mild to moderate symptoms who are at high risk of developing severe COVID-19. According to the COVID-19 Treatment Guidelines Panel (the Panel), the course of treatment consists of 300 mg nirmatrelvir plus 100 mg ritonavir orally twice daily for five consecutive days and should be promptly initiated within five days of symptom onset. This constitutes a strong recommendation with a moderate quality of evidence. The dose should be reduced to 150 mg nirmatrelvir plus 100 mg ritonavir twice daily in patients with eGFR ≥ 30 and <60 mL/min/1.73 m^2^. Paxlovid is not recommended for patients with eGFR < 30 mL/min/1.73 m^2^. However, based on limited data, a low-dose regimen has been proposed for patients with eGFR < 30 mL/min/1.73 m^2^ or undergoing hemodialysis. Paxlovid is also not recommended in patients with severe hepatic impairment (Child–Pugh Class C). It has also been authorized for emergency use in adolescents ≥ 12 years old weighing a minimum of 40 kg with mild to moderate COVID-19 who are at high risk of disease progression. Moreover, ritonavir-boosted nirmatrelvir is available through an FDA EUA for hospitalized patients with a diagnosis other than COVID-19 who have mild to moderate symptoms, provided that they are high-risk individuals and treatment initiation takes place within five days of symptom development [102]. Concerning pregnancy and lactation, the Panel recommends offering Paxlovid to this subset of the population based on a risk–benefit assessment. The most common side effects of this oral antiviral are dysgeusia, diarrhea, myalgia and hypertension [102].

On the other hand, the Panel makes a weak recommendation with moderate quality of evidence for the use of molnupiravir through an EUA by the FDA as an alternative therapeutic option in high-risk adult outpatients (≥18 years) with mild to moderate COVID-19 when ritonavir-boosted nirmatrelvir and remdesivir (intravenous drug) are not available, achievable to use or clinically relevant. The dosage is 800 mg orally twice daily for five days, and treatment should be started as soon as possible, within five days of symptom onset. Dosage adjustment is not necessary in cases of renal or hepatic impairment. According to the FDA, the risk of genotoxicity due to molnupiravir is low. The panel recommends using effective contraceptive methods in individuals who have sexual intercourse that may lead to conception, during and after treatment with molnupiravir. It also recommends against the use of molnupiravir during pregnancy unless there are no other therapeutic options and treatment is essential, according to expert opinion. Moreover, it advises against feeding an infant breast milk from a patient who receives molnupiravir for the duration of the treatment course and four days after the final dose. Finally, it may be used in hospitalized adults with a diagnosis other than COVID-19, mild to moderate symptoms and a high likelihood of disease progression. The most common adverse effects are diarrhea, nausea and dizziness, and no specific drug-to-drug interactions have been reported [103].

## 6. Future Perspectives on Oral Antiviral Treatments

Careful consideration of key checkpoints in the life cycle of SARS-CoV-2 (Figure 1) may lead to the formulation of further oral antiviral agents designed to impede host cell penetration, viral gene expression, replication and protein synthesis. Molnupiravir and Paxlovid emerged as the first oral antiviral treatments developed against SARS-CoV-2 in the rapidly evolving landscape of the COVID-19 pandemic, targeting viral gene replication and subsequent structural assembly, respectively.

Agents inhibiting the intracellular proteolytic cleavage facilitated by the SARS-CoV-2 papain-like protease (PL^PRO^) may induce an antiviral effect similar to the one instigated by the inhibitory properties of nirmatrelvir against 3CL^PRO^ [104]. Another promising area of focus might also lie in intercepting SARS-CoV-2 penetration of the respiratory mucosa by developing agents that disrupt the ACE2 cell surface receptor-viral spike protein interaction [105]. Lastly, transmembrane serine protease 2 (TMPRSS2) inhibition may also represent a possible therapeutic strategy against host cell infiltration by the virus [106]. Further clinical research is required to demonstrate the potential benefits of the aforementioned precision medicine targets in vitro, in vivo and in real-world clinical settings. 

## 7. Conclusions

Paxlovid and molnupiravir constitute the first effective oral antivirals against SARS-CoV-2, widely used during the Omicron era of the COVID-19 pandemic. These agents are currently recommended for patients with mild to moderate COVID-19 at increased risk of progressing to severe disease, as data from clinical trials and real-world studies have confirmed their efficacy in reducing hospitalization and mortality rates in various patient populations. Notably, a considerable portion of the worldwide population remains unvaccinated, and additionally, it has been demonstrated that vaccine-induced immunity declines over time. Oral antivirals account for a valuable addition to the therapeutic arsenal against SARS-CoV-2, with proven efficacy mainly in the outpatient setting. However, baseline patient comorbidities, vaccination status, viral load and timing of treatment initiation may significantly contribute to the apparent discrepancies in the efficacy of both agents, as demonstrated with clinical trials, real-world studies and meta-analyses. Further research is warranted to elucidate how these parameters affect the efficacy of oral antiviral treatment. Lastly, additional investigation is required to assess their comparative safety and efficacy in certain patient subgroups, like the immunocompromised, those with severe CKD, pregnant women, children, solid organ transplant recipients and those who experience COVID-19 rebound disease after oral antiviral treatment course conclusion.

## Data Availability

Not applicable.
